# Therapeutic Development Based on the Immunopathogenic Mechanisms of Psoriasis

**DOI:** 10.3390/pharmaceutics13071064

**Published:** 2021-07-11

**Authors:** Jen-Chih Tseng, Yung-Chi Chang, Chun-Ming Huang, Li-Chung Hsu, Tsung-Hsien Chuang

**Affiliations:** 1 Immunology Research Center, National Health Research Institutes, Zhunan, Miaoli 35053, Taiwan; mark0918@nhri.edu.tw; 2 Institute of Molecular Medicine, College of Medicine, National Taiwan University, Taipei 10002, Taiwan; lutti0909@gmail.com; 3 Department of Biomedical Sciences and Engineering, National Central University, Taoyuan 32001, Taiwan; chunmingsd@gmail.com; 4 Center of Precision Medicine, College of Medicine, National Taiwan University, Taipei 10002, Taiwan

**Keywords:** anti-psoriasis drugs, autoimmune, skin inflammation, Toll-like receptor, therapeutic antibody

## Abstract

Psoriasis, a complex inflammatory autoimmune skin disorder that affects 2–3% of the global population, is thought to be genetically predetermined and induced by environmental and immunological factors. In the past decades, basic and clinical studies have significantly expanded knowledge on the molecular, cellular, and immunological mechanisms underlying the pathogenesis of psoriasis. Based on these pathogenic mechanisms, the current disease model emphasizes the role of aberrant Th1 and Th17 responses. Th1 and Th17 immune responses are regulated by a complex network of different cytokines, including TNF-α, IL-17, and IL-23; signal transduction pathways downstream to the cytokine receptors; and various activated transcription factors, including NF-κB, interferon regulatory factors (IRFs), and signal transducer and activator of transcriptions (STATs). The biologics developed to specifically target the cytokines have achieved a better efficacy and safety for the systemic management of psoriasis compared with traditional treatments. Nevertheless, the current therapeutics can only alleviate the symptoms; there is still no cure for psoriasis. Therefore, the development of more effective, safe, and affordable therapeutics for psoriasis is important. In this review, we discussed the current trend of therapeutic development for psoriasis based on the recent discoveries in the immune modulation of the inflammatory response in psoriasis.

## 1. Introduction

The human skin covers the body. As it interfaces with the environment, it protects the body from heat, cold, and water loss. In addition, the skin plays a significant role in innate immunity by guarding the body against microbial infections. Psoriasis is an immune-mediated chronic inflammatory skin disease that affects the health and life quality of around 125 million people, or 2–3% of the global population [1,2,3,4]. This disease most commonly affects the extensor surfaces (including knees, elbows), the scalp, trunks, buttocks, limbs, and navels; the size of the affected area varies from small localized patches to most of the body, depending on the severity. In addition, about 50% psoriasis patients show nail (fingernail or toenail) involvement, and their clinical features include pittings, leukonychia, onycholysis, dyschromias, splinter hemorrhage, and nail bed hyperkeratosis. The disease may begin at any age, but it typically starts from the age of 15–25. Psoriatic inflammatory responses at the joints result in psoriatic arthritis, and up to 30–40% of individuals with psoriasis develop psoriasis arthritis between 30 and 50 years old [5,6,7,8].

Generally, psoriasis is thought to be a genetic disease induced by the interplay between genetic, internal, and external factors, including skin injuries, microbial infections, environmental influences, weather, stress, and immune disorders. It is characterized by erythematous and scaly plaques resulting from a marked thickening of the epidermis induced by the enhanced proliferation of keratinocytes, infiltration of leukocytes, and inflammation in the epidermis and dermis [9,10,11,12]. Sustained inflammation resulting from dysregulation of cellular signal transduction and overproduction of inflammatory cytokines is a recognized hallmark of psoriasis, which further causes the uncontrolled proliferation and dysfunctional differentiation of keratinocytes as well as the infiltration of leukocytes in psoriatic sites. The interplay among these molecular and cellular participants of psoriasis forms a feedback loop, further enhancing the extent and duration of psoriatic inflammation [13,14,15]. In this review, we summarized current knowledge on the immunopathogenic mechanisms of psoriatic inflammation and discussed the recent advances in the therapeutic development strategy of targeting the molecular factors and cellular signaling pathways involved in psoriasis.

## 2. Genetic and Environmental Factors in the Development of Psoriasis

While the exact etiology for the pathogenesis of psoriasis remains unclear, genetic factors are known to determine susceptibility to psoriasis. Pedigree analysis has demonstrated that children have an approximately 20% probability of developing psoriasis if one parent has psoriasis; the probability is about three times higher if both parents are afflicted. Twin studies have demonstrated that the disease concordance of psoriasis is two to three times higher in monozygotic twins than in the dizygotic twins [16,17]. Early linkage analyses of familial psoriasis have identified nine loci (*PRORS1*-9) associated with susceptibility to psoriasis. The linkage association of *PSORS1*, *PSORS2*, and *PSORS4* could be replicated independently in other studies, but other loci were not [18,19].

*PSORI1*, a strong heritable risk factor for psoriasis, accounts for 35–50% of the heritability. It maps to the major histocompatibility complex region on chromosome 6p21.3, which contains several candidate genes. There is a consensus that *HLA-Cw6* is most strongly associated with susceptibility to psoriasis. Given the role of *HLA-C* in presenting cellular antigen to CD8^+^ T cells, *HLA-Cw6* may have a high affinity with psoriasis autoantigens. So far, antimicrobial peptide LL-37 and a disintegrin-like and metalloprotease domain containing thrombospondin type 1 motif-like 5 (ADAMTSL5) have been shown to bind *HLA-Cw6*. LL-37 has been reported as a T cell autoantigen in psoriasis [20,21,22]. *PSORS2* is located on chromosome 17q. *CARD14*, which is identified in this locus, is responsible for psoriasis susceptibility. Caspase recruitment domain family member 14 (*CARD14*) is a scaffolding protein found to be produced at high levels in keratinocytes and involved in NF-κB signaling. Psoriasis-associated mutations in *CARD14* increase NF-κB activation, increasing the production of pro-inflammatory cytokines [23,24,25]. *PSORS4* is located on chromosome 1q21 spanning an epidermal differentiation cluster (EDC) region. Two EDC genes, namely *LCE3B* and *LEC3C*, encoding two late cornified envelope proteins, are strongly associated with psoriasis. Thus, there may be a link between skin barrier function and psoriasis [26,27,28].

Recent studies using genome-wide, exome-wide, and immune chip platforms have identified up to 63 psoriasis susceptibility loci. The candidate genes are often involved in different molecular pathways associated with psoriasis pathology. The candidate genes include genes involved in antigen presentation (e.g., *HLA-C*, *ERAP1*, and *ERAP2*), innate immune regulation (e.g., *IFIH1*, *DDX58*, *RNF114*, and *TYK2*), NF-κB regulation (e.g., *CARD14*, *TNFAIP3*, *TNIP1*, and *NFKBIA*), IL-23/IL-17 activation (e.g., *IL12Bp40*, *IL23Ap19*, *IL-23R*, and *TRAF3IP2*), and IL-36 activation (e.g., *IL36RN* and *AP1S3*) [19,29,30,31,32,33,34].

Environmental factors, including diet and obesity, smoking and alcohol intake, physical trauma, drug reactivity, and microbial infection, trigger psoriasis. Psoriasis lesions often develop on the extensor surfaces, such as elbows and knees, which endure more mechanical force. In addition, psoriasis more frequently occurs following a cutaneous injury, such as scratches, tattoos, and burns. Some drugs have been shown to induce and exacerbate psoriasis, including lithium, beta-blockers, tetracyclines, non-steroidal anti-inflammatory agents, imiquimod, angiotensin-converting enzyme (ACE) inhibitors, and calcium channel blockers. A considerable amount of data suggest that infections are a significant cause of psoriasis. Infection by *Streptococcus* has been associated with the occurrence of psoriasis. Moreover, the gut and skin microbiota, *Staphylococcus aureus* and *Malassezia* and *Candida albicans*, have been linked to the exacerbation of psoriasis [35,36,37,38,39,40,41]. Therefore, psoriasis is a multifactorial disease caused by the interaction between environmental and genetic factors.

## 3. Cellular and Molecular Immunomodulators in the Development of Psoriasis

Dysregulated immune response in skin lesions is another trigger of psoriasis, and it also plays a major role in maintaining development of psoriasis response. At the cellular level, psoriasis is characterized by the uncontrolled proliferation and differentiation of keratinocytes and infiltration of immune cells. At the molecular level, psoriasis is marked by the robust production of pro-inflammatory cytokines by these cells in the psoriatic sites [9,10,11,12].

Keratinocytes are the predominant cell type in the epidermis, where they form a barrier against environmental damage caused by heat, radiation, water loss, and microbial infections. In psoriasis, the proliferation of keratinocyte stem cells is dysregulated, rapidly increasing the proliferation of matured cells with reduced lipid and keratohyalin granules. The keratinocytes also interact with immune cells, mainly dendritic cells, monocytes/macrophages, and T cells, during the development of psoriasis [42,43].

Dendritic cells (DCs) are antigen-presenting cells that regulate the differentiation of naïve T cells into mature T cells. In psoriasis, there is an increased number of DCs in the epidermis and dermis. There are three major DC populations in the skin, namely Langerhans cells (LCs), plasmacytoid DCs (pDCs), and myeloid DCs (mDCs) [44,45]. LCs account for approximately 2–3% of the skin cells and are characterized by the expression of Langerin (CD207) and CD1a. The activation of LCs in the epidermis promotes their migration to draining lymph nodes where T cell activation is initiated. The cytokines produced by LCs contribute to the development of psoriasis by promoting Th17 and Th22 differentiations [46,47]. pDCs are characterized by HLA^−^DR^+^CD11c^−^CD123^+^BDCA-2^+^. In addition to TNF-α, IL-1β, and IL-6, pDCs produce a significant amount of interferon (IFN)-α, which is necessary to initiate the inflammatory response in psoriasis. Furthermore, the inhibition of IFN-α production by pDCs is observed to block the development of psoriasis in mouse models. pDCs are unique in their high expression of Toll-like receptor (TLR)7 and TLR9. These two TLRs are pattern recognition receptors that recognize the microbial RNA, unmethylated CpG-DNA, and self-RNA and self-DNA released in the psoriatic tissues [48,49,50]. Contrary to the role of pDCs in initiating psoriasis, the role of mDCs (CD11c^+^) lies in maintaining the development of psoriasis. Psoriatic inflammation significantly increases the number of mDCs in the lesions. mDCs derive from circulating precursors, migrate to the skin, and differentiate during the psoriatic inflammatory response. The dermal mDCs can be divided into two subpopulations, namely classical resident (CD11c^+^BDCA-1^+^) and inflammatory mDCs (CD11c^+^BDCA-1^−^). Resident mDCs participate in the in situ presentation of antigens to cutaneous T cells within the dermis. These mDCs in the psoriatic skin and normal skin have similar phenotypes and comparable numbers. Inflammatory mDCs, also known as TNF/iNOS-producing DCs (Tip-DCs), are present in high numbers in psoriatic skin lesions than in the normal skin and play an important role in the pathogenesis of psoriasis. Inflammatory mDCs produce TNF-α, IL-6, IL-12, IL-20, and IL-23, which are cytokines with critical roles in driving T cell differentiation into Th1 and Th17 phenotypes. Moreover, inflammatory mDCs produce iNOS, which generates NO [51,52,53,54].

Monocytes play an important role in the pathology of psoriasis as they are precursors of DCs and macrophages. Psoriatic monocytes have an increased capability of phagocytosis; they engulf low-density lipoprotein, leading to the robust production of inflammatory cytokines [55,56]. Macrophage infiltration at the dermal/epidermal boundary is a characteristic of psoriasis. Macrophages, which originate from circulating monocyte precursors, extravagate into target tissues and differentiate into different subsets of macrophages, depending on the microenvironment. The classically activated M1 macrophages and the alternatively activated M2 macrophages are the predominant subsets. M1 macrophage polarization is promoted by IFN-γ and microbial products, such as lipopolysaccharide (LPS). Conversely, M2 macrophages are polarized by stimuli, such as macrophage colony-stimulating factor (M-CSF), IL-4, IL-10, and IL-13. M1 macrophages are involved in inflammatory responses by producing chemokine ligands and pro-inflammatory cytokines, including TNF-α, IL-1, IL-6, IL-12, and type I IFNs [57,58,59]. The endogenous ligands of TLR7 and TLR9, including the self-RNA and self-DNA released in psoriatic lesions, have been demonstrated to promote the polarization of macrophages into the M1 phenotype through TLR activation [60]. The role of macrophages in the pathology of psoriasis has been demonstrated in various animal studies. Macrophages can be involved in the amplification of inflammatory responses at the psoriatic lesions at different stages of psoriasis development. The depletions of macrophages by clodronate liposomes and colony stimulating factor 1 receptor (CSF-1R) inhibitor were reported to inhibit the inflammatory responses in animals with imiquimod and IL-23-induced psoriasis. Imiquimod initiates psoriatic inflammation by activating TLR7 in pDCs, whereas IL-23 enhances inflammation in the psoriatic skin by promoting Th17 responses [60,61,62].

B cells are well known for their role in host response to microbial infection by producing antibodies. Nevertheless, there is a subset of B cells named regulatory B cells (Bregs) known to suppress immune functions by production of IL-10. Partially due to the rare appearance of B cells in psoriatic lesions, the role of these cells in the development of psoriasis is still unclear. However, studies from genetically modified mice lacking IL-10-producing B cells have showed that loss of Breg function can result in chronic inflammation. The levels of Breg are reduced in patients with psoriasis. Further, treatment of psoriasis patients with IL-10 was shown to improve the disease symptom [63,64,65].

The role of T cells in psoriasis has been well defined. Both CD4^+^ (regulatory, helper) T cells and CD8^+^ (effector, cytotoxic) T cells play an important role in the development of psoriasis. In psoriasis animal models, the injection of CD4^+^, but not CD8^+^, T cells from psoriatic patients induced psoriasis in SCID mice. This CD4^+^ T cell-driven process proceeds to CD8^+^ T cell recruitment and activation, as the development of psoriasis in animal models is eliminated by inhibiting CD8^+^, but not CD4^+^, T cells [66,67]. Based on the cytokine production profiles, the CD4^+^ T helper (Th) cells can be divided into several subsets, namely regulatory T (Treg), Th1, Th2, and Th17. Treg cells are immunosuppressive and generally suppress the proliferation of effector T cells by secreting immunosuppressive cytokines, including transforming growth factor beta (TGF-β), IL-10, and IL-35. Antigen-activated T cells produce IL-2 acting on the IL-2 receptors on Treg cells, alerting them of the high T cell activity in the environment so that Treg cells will generate a suppressive response. Tregs are characterized by the expression of CD4, CD25, and forkhead box P3 (FOXP3). Th1 and Th2 cells are two canonical subsets of T cells. Th1 cells are characterized by the expression of transcription factor T-bet and the release of IFN-γ, TNF-α, and IL-12. Th2 cells express transcription factor GATA-binding protein 3 (GATA-3) and produce IL-4, IL-5, and IL-13. Differentiation of Th17 cells is driven by IL-1β, IL-6, and IL-23. Th17 cells produce signature retineic-acid-receptor-related orphan nuclear receptor gamma T (RORγT) and release IL-17, TNF-α, IL-21, IL-22, and IL-26. Worth to note is that these cells are the major source of IL-17, which plays a crucial role in development of psoriasis. IL-17 stimulates keratinocytes to produce antimicrobial peptides and proinflammatory cytokines, which in turn promote dendritic cells to produce cytokines including TNF-α, IL-1β, IL-6, IL-12, and IL-23; this results in T cell differentiation and activation, forming a positive feedback loop to drive psoriatic inflammation [66,67,68,69,70].

## 4. Major Cellular Signal Transduction Pathways Involved in the Development of Psoriasis

The cellular and molecular immunomodulators in the development of psoriasis are integrated into several major signal transduction pathways; as a result, they activate transcription factors, including NF-κB, IRFs, and STATs, in cells.

**Figure 1:**
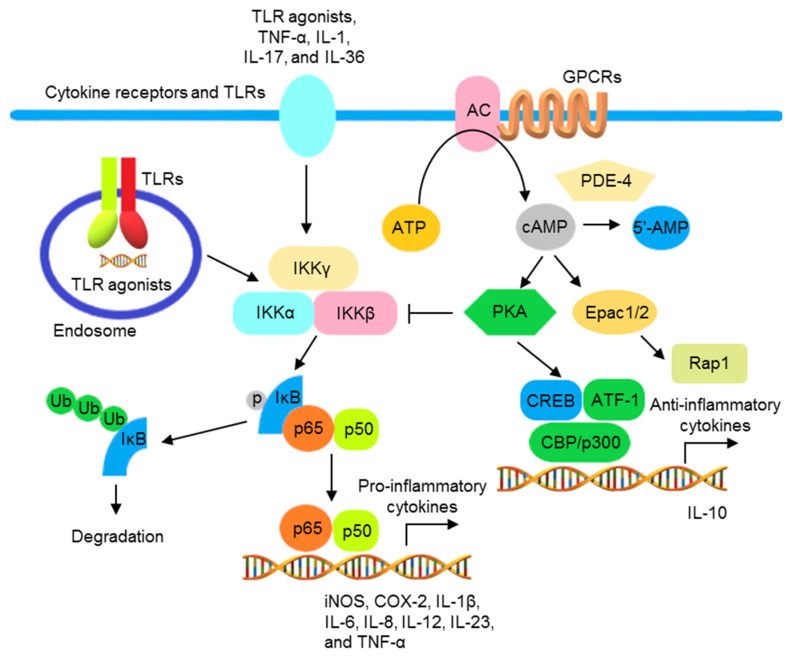
The schematic view of the inflammatory responses mediated by NF-κB activation and regulated by cAMP. Signal transductions activated by TLR agonists, TNF-α, IL-1, IL-17, and IL-36, resulting in the phosphorylation of IKKβ and formation of IKK complex. The activated IKK complex promotes the phosphorylation, ubiquitination, and subsequent proteasomal degradation of IκB, resulting in the translocation of NF-κB to the nucleus to promote the transcription of various NF-κB-controlled inflammatory cytokine genes. AC, activated by GPCRs, increases intracellular cAMP. The accumulated cAMP activates PKA, interfering with IκB ubiquitination and degradation by blocking the formation of IKK complex. PKA also activates CREB/ATF1 and increases the transcription of anti-inflammatory cytokine genes. Moreover, cAMP activates exchange protein directly activated by cAMP (Epac), regulating Ras-related protein (Rap)1 and reducing inflammatory responses. PDE 4 degrades cAMP into 5′-AMP that reduces the inhibition of the NF-κB activation by cAMP.

TLR agonists, IL-1, IL-17, IL-36, and TNF-α activate the NF-κB signaling pathway to promote transcription of various genes encoding inflammatory mediators and cytokines, including iNOS, cyclooxygenase-2 (COX-2), IL-1β, IL-6, IL-8, IL-12, IL-23, and TNF-α [71,72]. NF-κB activation is inhibited by cAMP (the second messenger downstream to G protein-coupled receptors (GPCRs)) in most immune cells, including lymphocytes, monocytes/macrophages, and epithelial cells. Adenylate cyclase (AC), activated by GPCRs, catalyzes the synthesis of cAMP from ATP. The accumulated cAMP activates protein kinase A (PKA) and blocks IKK and NF-κB activation. In addition, the phosphorylation of cAMP response element binding protein (CREB) by activated PKA increases the expression of IκB and anti-inflammatory cytokines. Conversely, cAMP can be hydrolyzed into 5′- adenosine monophosphate (5′-AMP) by phosphodiesterase 4 (PDE4), increasing NF-κB activation (Figure 1) [73,74,75].

**Figure 2:**
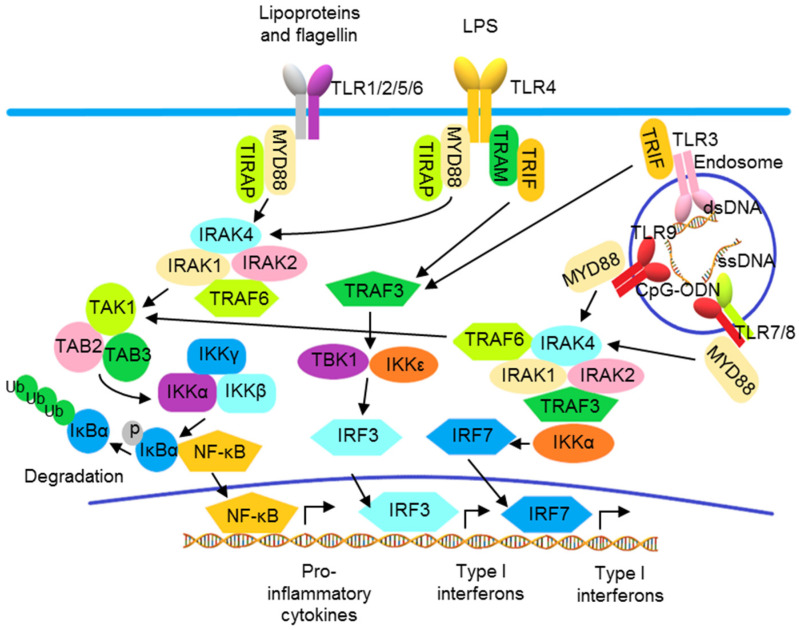
Toll-like receptor signaling pathways. TLRs 1, 2, 4, 5, and 6 localize to the cell surface, and TLRs 3, 7, 8, and 9 localize to intracellular vesicles, such as endosomes, where they recognize their ligands, including exogenous pathogen-associated molecular patterns (PAMPs) and endogenous damage-associated molecular patterns (DAMPs). The TLRs use the adaptor proteins of the MyD88 family, including MyD88, TRIF, TIRAP, and TRAM, to initiate downstream signaling pathways, leading to the activation of various transcription factors, including IRF3/7 and NF-κB, and the production of type I interferons and pro-inflammatory cytokines.

In addition to the activation of NF-κB to produce inflammatory cytokines, TLR also activates IRFs to produce type I IFNs. Except for TLR3, all TLRs utilize MyD88, which is also a molecule for the downstream signaling to IL-1 and IL-36 receptors. Upon activation, TLR binds MyD88, recruiting IL-1R-associated kinases (IRAKs) and TNF receptor-associated factors (TRAFs) to form a signalosome leading to the IKK complex-mediated NF-κB activation. TLR3 and TLR4 recruit TRIF to activate NF-κB and IRF3 through TRAF6 and TBK1 and the IKKε/IKK*i* complex, respectively (Figure 2) [76,77,78]. In pDCs, following the activation of endosomal TLRs including TLRs 7, 8, and 9, IRF7 is phosphorylated after activation by the MyD88 signalosome and then translocates to the nucleus to induce transcription of the genes encoding type I IFNs (Figure 2) [79]. Other than the utilization of adaptor molecules, cellular location also determines the activation of TLR signaling. The engagement of TLR9 by its ligand in distinct endosomal compartments of pDCs can result in the differential activations of NF-κB and IRF7 pathways [80,81].

**Figure 3:**
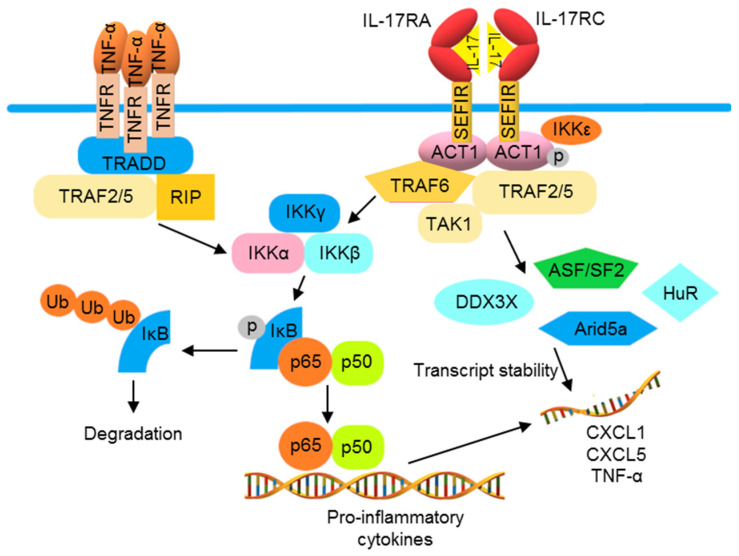
TNF-α and IL-17 cooperate to promote the production of pro-inflammatory cytokines. The activation of TNFR induces the production of pro-inflammatory cytokines by recruiting TRADD, TTRAF2 and 5, and RIP1 to the receptor, thus activating the IKK complex and NF-κB. IL-17 receptor (IL-17R) signaling can also induce NF-κB via ACT1 and TRAF6. Furthermore, IL-17R initiates mRNA stabilization signaling through the IKKε-mediated phosphorylation of ACT1, which binds to TRAF2 and 5 to activate mRNA binding proteins to increase mRNA instability. The combination of TNFR and IL-17R activations often results in a synergistic inflammation that can be partially explained by increased mRNA expression and stabilization.

Compared with the TLRs and receptors of IL-1 family proteins, TNF-α and IL-17 receptors utilize distinct signaling molecules for NF-κB activation. The trimeric TNF receptor (TNFR) complex recruits tumor necrosis factor receptor 1-associated death domain (TRADD), TRAF2, and TRAF5 for binding with receptor-interacting serine/threonine-protein kinase 1 (RIPK1) to activate NF-κB [82,83,84]. The IL-17 cytokine family consists of six members, namely IL-17A to IL-17F, which are produced by different cell types. IL-17A, often known as IL-17, is the best-characterized member. IL-17 binds to an IL-17 receptor (IL-17R)A/IL-17RC heterodimeric receptor, recruiting an ACT1 (encoded by the gene *TRAF3IP2*) adaptor protein, leading to activation of the downstream signaling pathway that involves TRAF6 and TAK1, thus activating NF-κB [85,86]. A second pathway diverges from ACT1, connecting IL-17 activation to mRNA stabilization. Through an ACT1-, TRAF2-, and TRAF5-dependent mechanism, IL-17 signaling can activate some RNA-binding proteins (RBPs). These RBPs are involved in the IL-17-induced stabilization of target mRNAs including C-X-C motif ligand CXCL1, CXCL5, and TNF-α. IL-17 and TNF-α have been shown to synergistically amplify inflammatory responses in psoriatic lesions, likely due to the increased stability of TNF-α-induced mRNA transcripts of inflammatory mediators by this mRNA stabilization function of IL-17 activation (Figure 3) [87,88,89].

**Figure 4:**
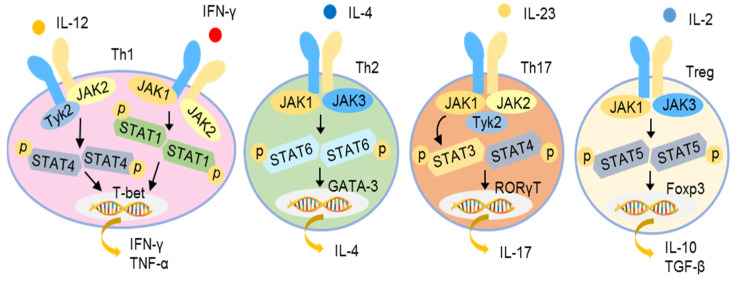
JAK-STAT signaling pathways of different cytokines for initiation of T helper responses. Different combinations of JAKs are activated by different cytokine receptors to promote the phosphorylation and dimerization of different STAT proteins as illustrated. The dimerized STATs translocate to the nucleus to activate the transcription of target genes and initiate different Th responses as illustrated.

Various cytokines, such as type I IFNs, IFN-γ, IL-2, IL-4, IL-6, IL-10, IL-12, IL-21, IL-22, and IL-23, activate cells through the so-called type I and II receptors. Upon ligation, the type I and II cytokine receptors become oligomerized, leading to the recruitment and autophosphorylation of JAKs and the subsequent activation of STATs, followed by their nuclear translocation to modulate gene expression. Four JAKs, namely JAK1, JAK2, JAK3, and tyrosine kinase 2 (TYK2), have been identified. The STAT family consists of seven members, namely STAT1, STAT2, STAT3, STAT4, STAT5A, STAT5B, and STAT6 [90,91]. Different cytokine receptors utilize different JAK and STAT pairs for signal transduction. For example, IFN-γ receptor induces JAK1/JAK2 activation and then promotes STAT1/STAT1 homodimerization, while IL-12 receptor uses JAK2/TYK2 to activate STAT4/STAT4 homodimerization to trigger a Th1 immune response. The engagement of the IL-4 receptor results in the dimerization of JAK1/TYK3, activating STAT6/STAT6 homodimerization to promote a Th2 response. The activation of the IL-23 receptor leads the recruitment of a JAK1/JAK2/TYK2 complex, activating STAT3/STAT4 dimerization to induce a Th17 response. Similarly, IL-2 receptor activation utilizes JAK1/JAK2 to activate STAT5/STAT5 nuclear translocation to induce a Th2 response (Figure 4) [92,93,94].

## 5. Current Model for the Pathogenesis of Psoriasis

**Figure 5:**
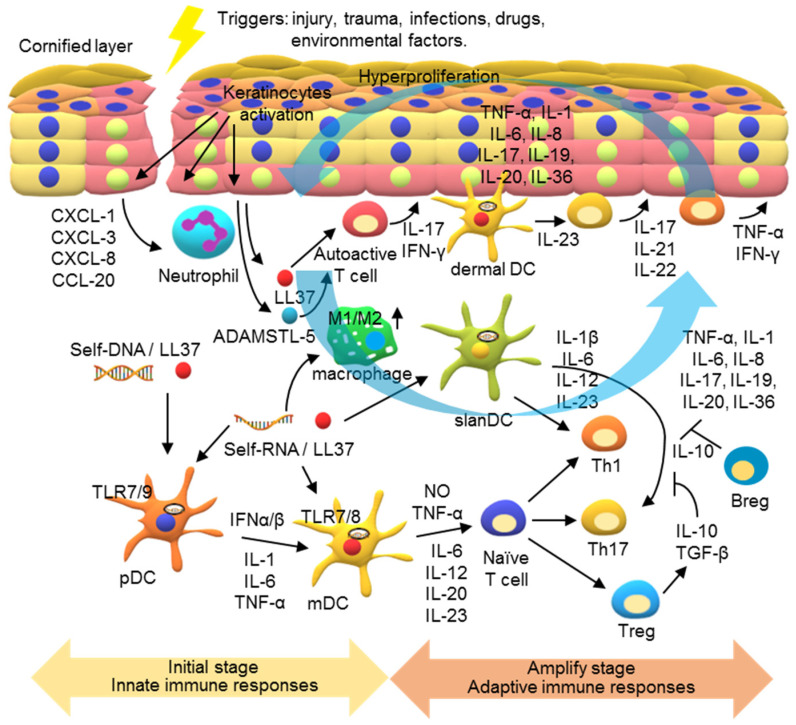
A model for the pathogenesis of psoriasis. In the initial stage of psoriasis, environmental stress, such as skin damage and infection, causes the release of damage-associated molecular patterns (DAMPs), such as self-RNA and self-DNA. Antimicrobial peptides (AMPs), such as LL-37 released from keratinocytes, can bind to these self-nucleic acids, inducing innate immune responses in the psoriatic lesions. The LL-37/DNA complex activates TLR7 and TLR9 in plasmacytoid dendritic cells (pDCs) to release pro-inflammatory cytokines and type I IFNs, which activate the maturation of myeloid dendritic cells (mDCs). LL37/RNA complexes can also activate TLR8 in mDCs to produce IL-12 and IL-23. Moreover, the polarization of macrophages into the M1 subset can be activated to produce pro-inflammation cytokines at this stage. In the development phase of psoriasis, adaptive immune responses are activated and psoriatic inflammation is amplified. The cytokines in the psoriatic lesions activate Th1 and Th17 cells for production of various cytokines to contribute to the inflammatory milieu and act on keratinocytes. The keratinocytes are then activated to produce inflammatory cytokines, chemokines, and AMPs to recruit leukocyte infiltrates and activations, forming a self-amplifying feedback loop in the psoriatic inflammatory response.

Under normal conditions, the signal transductions in skin cells are well controlled to maintain the homeostasis of the immune system for host defense and preventing disorders. However, under pathogenic conditions, massive inflammatory cytokines and excessive immune responses can be generated due to overactivation of these signaling transductions. Currently, a generally accepted model for the pathogenesis of psoriasis involves dysregulation of immune responses in two stages. In the initial stage, innate immune responses are triggered in the psoriatic sites (Figure 5). The external stimuli stimulate the release of antimicrobial peptides (AMPs) from keratinocytes and self-nucleic acids from dying cells in the epidermis, activating the innate immune responses in the DCs.

DC activation is mediated by endosomal TLRs. These TLR genes are differentially expressed in different subsets of DCs. pDCs mainly produce TLR7 and TLR9; contrarily, mDCs produce TLR7 and TLR8. Released AMP, such as LL37, can form a complex with self-DNA and self-RNA to facilitate their entrance into the cells. Activating the TLR7- and TLR9 in pDCs by the LL37/self-nucleic acids results in production of pro-inflammatory cytokines, including IL-1, IL-6, TNF-α, and type I IFNs. These pDCs produce cytokines, particularly type I IFNs, in turn activating mDCs. In addition, LL37/self-RNA complexes can directly activate mDCs via TLR7 and TLR8, turning them into highly inflammatory dermal mDCs that produce NO, TNF-α, IL-6, IL-12, IL-20, and IL-23. These cytokines modulate T cell proliferation and differentiation into various subsets, including Th1 and Th17 [95,96,97,98,99,100]. In addition, a subset of slanDCs (6-sulfo LacNAc) is inflammatory dermal DCs in psoriasis. slanDCs respond particularly well to the LL37/self-RNA complexes, producing high levels of IL-1ß, IL-6, IL-12, and IL-23 for Th1 and Th17 programming [101]. Another mechanism underlying psoriasis involves autoactive T cells that produce IFN-γ and IL-17. These lymphocytes are reactive to self-antigens, such as LL-37, ADAMSTL5, and nanolipids, as suggested by the presence of the autoactive T cells specific to LL-37 and ADAMSTL5 in patients with psoriasis. Nevertheless, no monoclonal expansion of the autoactive T cells in the psoriatic lesions has yet been characterized. How the self-antigens and autoactive T cells initiate psoriasis remains to be studied [102,103,104].

Following the first phase of dendritic cell-mediated innate immune activation, the activated T cells mediate adaptive immune responses to amplify psoriatic inflammation (Figure 5). The activation of DCs leads to a sustained T cell response that includes reorganized dermal T cell infiltration and formation of interactive DCs and T cell clusters in the inflammatory psoriatic milieu [105,106]. Furthermore, most of the infiltrating dermal DCs produce IL-23 to maintain the IL-17-generating T cells. Th17 cytokines, mainly IL-17, IL-21, and IL-22, and Th1 cytokines, TNF-α and IFN-γ, in the inflammatory milieu of psoriatic lesions activate keratinocyte proliferation in the epidermis [107,108,109,110].

Keratinocyte activation plays a major role in a feedback loop in the IL-23/IL-17-driven inflammation [42,43,111,112]. IL-17A’s synergy with other cytokines, such as TNF-α and IL-22, stimulates the production of AMPs, inflammatory cytokines, chemokines, and chemoattractants in keratinocytes. AMPs include LL-37, the S100A family of protein, β-defensins, and lipocalin-2. LL-37, shown to function as a self-antigen, facilitates DC activations mediated by TLRs 7, 8, and 9 [49,50,85,95]. Cytokines, including TNF-α, IL-1, IL-6, IL-8, IL-17, IL-19, IL-20, and IL-36, act as paracrine and autocrine mediators to drive the psoriatic inflammatory loop. IL-19 and IL-20 activate keratinocyte hyperproliferation, leading to epidermal hyperplasia. IL-36 has been shown to act on keratinocytes and immune cells to induce a robust inflammatory response in the psoriatic lesions [113,114,115,116]. Chemokines include C-C motif ligand (CCL)20, which plays an important role in recruiting C-C motif chemokine receptor (CCR)6^+^ cells, such as IL-17-producing T cells and some DCs. Chemoattractants including CXCL1, CXCL3, and CXCL8 attract neutrophils and sustain their activation and survival, contributing to the production of AMPs and pro-inflammatory cytokines [117,118,119]. In addition, emerging evidence has indicated macrophage recruitment and activation in psoriatic lesions; the ratio of M1/M2 macrophages in patients with severe psoriasis is high. LL-37/self-nucleic acid complexes and cytokines, such as IFN-γ, can promote macrophage polarization into the M1 subset. Th1/Th17 cells and keratinocytes can be activated by inflammatory cytokines, including TNF-α, IFN-γ, IL-17, and IL-23, produced by M1 macrophages and integral to the IL-23/IL-17 axis [60,61,62,120,121]. Together, these events result in a self-amplifying feedback inflammatory response in the psoriatic sites and cause chronic psoriatic inflammation.

## 6. Mechanisms of the Current Therapies for Psoriasis

Psoriasis is a chronic disease that often requires long-term treatment. Various therapeutic options, including topical therapies, phototherapies, and systemic therapies, are available for the management of psoriasis. The choice of therapy is based on the severity of the disease. Generally, topical treatments are preferred for mild psoriasis to avoid unwanted systemic effects on the patient. Topical agents include coal tar, salicylic acid, retinoids, vitamin D analogs, anthralin, calcineurin inhibitors, and corticosteroids. Phototherapy, which suppresses the immune response at the psoriatic sites, is mainly for patients with moderate-to-severe psoriasis or those who do not respond to topical treatments. The phototherapies that are usually recommended are ultraviolet (UV)-A therapy with psoralen and UV-B therapy. The common side effects of phototherapy include itching and burning; although skin cancer is rare, it remains a risk. Conversely, systemic drugs are used to treat moderate-to-severe psoriasis [9,10,11,12,122,123]. Systemic drugs can be classified into small-molecule drugs and biologics. The commonly used small-molecule drugs include methotrexate, cyclosporine A, acitretin, fumaric acid esters, and apremilast. All of these drugs are orally administered; methotrexate is also available for subcutaneous administration (Table 1).

Methotrexate, an anti-inflammatory drug, has been utilized to manage psoriasis for more than 50 years. It increases adenosine production and inhibits lymphocyte function [124,125,126]. Cyclosporine was a key medication for severe psoriasis until the development of biologics. However, it remains important for psoriasis treatment. As a polypeptide inhibitor, it suppresses calcineurin, a phosphatase critical in T cell signaling. The activation of the T cell receptor by antigen increases the intracellular calcium level, activating calcineurin, dephosphorylating cytoplasmic NFTAc (nuclear factor of activated T cells), causing NFTAc to translocate to the nucleus, and inducing transcription of several cytokines, such as IL-2. Thus, cyclosporine acts as an immune suppressant by blocking calcineurin-mediated NFTAc dephosphorylation, cytokine production, and T cell activation [127,128,129]. Acitretin is a synthetic retinoid. Retinoids are vitamin A related compounds. They activate retinoic acid receptors and retinoid X receptors, causing the activated nuclear receptors to bind to the retinoid hormone response elements in the promoter region of the target genes and initiating gene transcriptions. As a result, acitretin improves the symptoms of psoriasis by normalizing the proliferation of keratinocytes and reducing the production of inflammatory cytokines [130,131,132]. Fumaric acid esters were first utilized for systemic psoriasis treatment in 1959 in Germany. It is one of the most commonly used treatments for psoriasis in Germany and is increasingly used as an unlicensed treatment in several other European countries. Fumaric acid esters exhibit anti-oxidation, immunomodulation, and anti-inflammatory properties. The drug’s exact mechanism remains unclear; however, it likely blocks NF-κB activation, impedes dendritic cell maturation, and inhibits inflammatory T cell responses [133,134,135]. Apremilast, a PDE4 inhibitor, inhibits the hydrolyzation of cAMP. The stabilization of cAMP by apremilast alleviates psoriatic inflammation by suppressing NF-κB activation and reducing the expression of the genes encoding inflammatory cytokines. PDE4 is produced in most immune cells, including monocytes/macrophages, granulocytes, and lymphocytes, and other cells, such as epithelial cells. Therefore, apremilast exhibits a broad anti-inflammatory effect [136,137,138]. The five systemic small-molecule drugs mentioned above vary in efficacy, usually achieving a 75% reduction from the baseline Psoriasis Area and Severity Index score (PASI75) at week 12 for 30–65% of patients with psoriasis [122,123].

Over the past decades, the development of biologics, mostly monoclonal antibodies, has significantly increased our ability to manage psoriasis. These biologics block the initiation and progression of psoriatic inflammation by specifically targeting key modulators in the psoriatic immune response [122,123]. Currently, there are 11 biologics available for the systemic treatment of moderate-to-severe psoriasis. They antagonize TNF-α, IL-17, the p19 subunit of IL-23, the p40 subunit of IL-12, and IL-23 (Table 1). TNF-α inhibitors are the first-generation biologics for psoriasis. Etanercept, infliximab, adalimumab, and certolizumab pegol are included in this category. Etanercept is unique among the biologics; it is not a monoclonal antibody but a fusion protein of TNFR2 and the Fc region of IgG1. Infliximab is a chimeric monoclonal antibody. Adalimumab is a fully human monoclonal antibody. Both infliximab and adalimumab bind to both soluble and membrane-bound TNF-α and neutralize its activity. Certolizumab pegol is a polyethylene glycol-conjugated human monoclonal antibody against TNF-α. Pegylation increases the half-life and decreases the immunogenicity of the antibody [139,140,141,142,143]. In addition to the TNF-α inhibitors, three anti-IL-17 biologics, namely, secukinumab, ixekizumab, and brodalumab, are used for psoriasis. Both secukinumab and ixekizumab specifically target IL-17A, whereas brodalumab targets IL-17RA. IL-17RA is involved in the signal transduction by several members of the IL-17 family. Thus, by targeting IL-17RA, brodalumab inhibits the inflammatory responses initiated by IL-17A, IL-17C, IL-17F, and IL-17E (IL-25) [144,145,146,147]. So far, four anti-IL-23 biologics, tildrakizumab, guselkumab, risankizumab, and ustekinumab, have been approved for use. Tildrakizumab, guselkumab, and risankizumab target the p19 subunit of the IL-23. In contrast, ustekinumab targets the p40 subunit shared by IL-12 and IL-23. Except for infliximab, which is administered via intravenous infusion, all the biologics currently used for psoriasis are administered subcutaneously [148,149,150,151,152]. Compared with the small-molecule drugs, these biologics demonstrate an improved efficacy. Approximately 55–80% of the patients treated with the TNF-α inhibitors and 80–90% of the patients treated with the IL-17 inhibitors reach PASI75 at week 12. Around 64% of the patients treated with tildrakizumab and ustekinumab achieve PASI75, and about 73% of the patients treated with guselkumab and risankizumab achieve PASI90 at week 12 [122,123]. The effectiveness of psoriasis treatment by blocking IL-17 also suggests a critical role of the Th17 response in development of psoriatic inflammation.

## 7. Strategies for the Development of Low-Cost and Effective Novel Psoriasis Therapeutics

Different forms or analogs of fumaric acid esters and apremilast and different monoclonal antibodies against TNF-α, IL-17, and IL-23 are also in the stage of clinical development for psoriasis treatment (Table 2). In addition, biologics that target other modulators of the psoriatic inflammatory response, including the inhibitors of IL-36R and IL-1 and activators of IL-2R, cytotoxic T-lymphocyte-associated protein 4 (CTLA-4), and programmed cell death protein 1 (PD-1), are in different stages of clinical testing (Table 2) [153,154,155,156,157,158,159,160,161,162,163].

IL-36 is a pro-inflammatory cytokine of the IL-1 cytokine family. Both IL-36 and IL-1 play an important role in the activation of the Th17 response. Spesolimab (BI 655130) and imsidolimab (ANB019) are monoclonal antibodies that target IL-36R. Anakinra is a recombinant form of human IL-1R antagonist (IL-1Ra). Gevokizumab and canakinumab are monoclonal antibodies against IL-1β. These inhibitors block the IL-36/IL-17 and IL-1/IL-17 axis of the psoriatic inflammatory response [153,154,155,156,157,158]. IL-2 plays a crucial role in promoting the differentiation of immature T cells into Treg cells that suppress the function of other T cell subsets. Therefore, the potentiation of Treg functions can be a strategy for the treatment of autoimmune diseases. LY 3471851 (NKTR 358) is a polyethylene glycol (PEG) conjugate of recombinant IL-2. CC 92252 (DEL 106) is an IL-2 mutein Fc fusion protein. These two biologics are agonists to IL-2R [159,160]. CTLA-4 and PD-1 are immune checkpoint regulators preventing inflammatory or autoimmune disorders by maintaining the level of T cell activation within a normal range. Abatacept, an Fc fusion protein of CTLA-4 ectodomain, can inhibit the co-stimulation of T cells by binding to CD80/CD86. Abatacept is used to treat rheumatoid arthritis and has been approved for psoriatic arthritis since 2017. It was clinically investigated for psoriasis; however, no result was posted. LY 3462817 is a monoclonal antibody agonist of PD-1 in the clinical development for psoriasis [161,162,163].

The approved biologic drugs have substantially improved the effectiveness of psoriasis management; however, they have some deficiencies. First, the biologics have side effects, the most common of which include an increased risk of infections and injection site reactions. Second, these biologics often require continuous administration. Third, a subset of patients does not respond to these biologics. Fourth, biologics are expensive and may not be affordable for all patients. Therefore, the development of low-cost and effective drugs, such as biosimilars, for the treatment of psoriasis is important. A biosimilar is a biologic product highly similar to an approved biologic; it has no clinically meaningful differences in safety, purity, and effectiveness from the reference product. It is desirable to develop low-cost biosimilars after the expiration of the patents for a biologic drug, which are more affordable [164,165,166]. The biosimilars of anti-TNF-α biologics are already available. Two etanercept biosimilars, eight adalimumab biosimilars, and four infliximab biosimilars have been approved for the treatment of psoriasis by the US Food and Drug Administration (FDA) and/or the European Medicines Agency. The price of these biosimilars is around 5–30% lower than their originator biologics. As a lower-cost alternative, these biosimilars reduce the economic burden on patients. This, plus the health insurance plans of different countries, enables the use of affordable biologics for a high percentage of psoriasis patients in many areas [166,167,168]. Other versions of anti-TNF-α biosimilars are already in the stage of clinical testing (Table 2).

Another approach to developing low-cost and effective drugs for psoriasis is to identify small-molecule drugs that target novel modulators in the psoriatic inflammatory signaling pathways. Several categories of drug candidates that target different signaling molecules are under development (Table 2). The regulators of Th17 responses are major targets for small-molecule drugs. JAK-STAT signaling is crucial for the activation of Th17 responses. The JAK inhibitors investigated for psoriasis include small-molecule drugs tofacitinib and baricitinib. Tofacitinib is an oral JAK1 and JAK3 inhibitor approved for rheumatoid arthritis and psoriatic arthritis. Baricitinib is an oral inhibitor of JAK1 and JAK2. It has already been approved for rheumatoid arthritis. Both tofacitinib and baricitinib are in phase 2 testing for psoriasis. BMS-986165 and PF-06826647 are two small-molecule TYK2 inhibitors. BMS-986165 is in phase 3 testing for psoriatic arthritis and psoriasis, whereas PF-06826647 is in phase 2 development [169,170,171,172,173,174]. RORγT, a transcription factor expressed in Th17 cells, plays a crucial role in Th17 differentiation and controls the expression of Th17 cytokine genes. VTP-43742, JET-451, ABBV-157, and PF-06763809 are small-molecule inhibitors of RORγT investigated in the clinical trials for psoriasis. However, no RORγT inhibitor has reached phase 3 so far [175,176,177,178].

NF-κB is an important transcription factor for the regulation of pro-inflammatory cytokine gene expressions. Modulators that regulate NF-κB are also major drug targets for psoriasis [72]. CF101 is a small-molecule agonist for the A3 adenosine receptor (A3AR), which is a G protein-coupled receptor. Activation of the A3AR by CF101 induces G protein inhibitory pathways, decreasing the level and activity of PKB/Akt and deregulating NF-κB. CF101 is in a phase 3 study for psoriasis. RIP1K is an adapter protein in TNF-α signaling and a regulator of NF-κB activation. GSK2982772, a small-molecule inhibitor of RIPK1, has been investigated for the treatment of ulcerative colitis, rheumatoid arthritis, and psoriasis. IMO-8400, an antagonist of TLR7, 8, and 9, blocks NF-κB activation by competing against the ligand binding of LL37/self-DNA and LL37/self-RNA complexes to these TLRs. The results from a phase 2 study indicated that 45% of the patients receiving IMO-8400 at any dose level achieved PASI50 at week 12 compared with 14% of the patients receiving placebo [179,180,181].

Other small-molecule drug targets investigated for psoriasis include heat shock protein (HSP)90, aryl hydrocarbon receptor (AhR), sphingtosine-1-phosphate receptor 1 (S1PR1), and Rho-associated kinase 2 (ROCK2). HSP90 is a chaperone protein that binds to many different client proteins and increases their stability. Some of the client proteins are involved in the regulation of psoriatic inflammatory responses. HSP90 inhibitors have been shown to significantly inhibit the IL-17A- and TNFα-induced inflammatory responses. CUDC-305 (Debio 0932), an HSP90 inhibitor, is in phase 1 trial for psoriasis [182,183]. AhR is a ligand-dependent transcription factor regulating gene expression in various cell types, including immune and epithelial cells. The activation of AhR by its agonist tapinarof has been shown to downregulate the expression of genes encoding pro-inflammatory cytokines, including IL-17, and promote skin barrier normalization. DMVT-505 (benvitimod), a 1% tapinarof cream, is currently in phase 3 development [184,185,186]. S1PR1 is a G protein-coupled receptor. Its signaling is vital in controlling the maturation, migration, and trafficking of lymphocytes. Ponesimod, an oral antagonist of S1PR1, prevents lymphocytes from leaving lymph nodes, thus reducing the number of peripheral lymphocytes and preventing their infiltration into the tissues. In a phase 2 study, 46% and 48% of the patients receiving 20 and 40 mg of ponesimod, respectively, achieved PASI75 at week 12 compared with the 13% of the placebo patients [187,188]. KD025 is an oral ROCK2 inhibitor that can reduce Th17 immune responses. In a phase 2 study, 71% of the patients taking 200 mg of KD205 twice daily achieved PASI50 at week 12. Therefore, targeting ROCK2 has a significant potential as a psoriasis therapy [189,190].

## 8. Conclusions

Psoriasis places a tremendous physical and psychological burden on patients. In many previous studies, most of the mechanisms underlying the pathogenesis of psoriasis have been revealed. There is a consensus that psoriasis is genetically predetermined, and it can be triggered by environmental and immune factors. Sustained inflammation in the psoriatic sites mainly contributes to the severity of psoriasis and difficulty in treatment. Traditionally, broad-acting anti-inflammatory and immune-suppressive therapies, such as methotrexate, cyclosporine, acitretin, and phototherapies, are used for the treatment of psoriasis. However, the limited efficacy and adverse effects of these drugs render them unsuitable for many patients. Based on our understanding of the immunological control of psoriatic inflammation, biologics targeting the TNF-α, IL-17, and IL-23 signaling pathways have been developed to treat patients with moderate-to-severe psoriasis. Compared with traditional therapeutics, biologics are more effective and have fewer side effects. However, they are much more expensive and thus inaccessible to many patients because the access to biologic therapies for psoriasis differs from country to country. In some countries, the biologics are covered by insurance, while in others they are not covered.

The development of more affordable, safer, and more effective systemic drugs for patients with moderate-to-severe psoriasis is important. One approach for this is the development of biosimilars. In addition, the study of the mechanisms underlying the immunomodulation of psoriatic inflammation demonstrates that more molecules can be drug targets for psoriasis. Novel biologics and small-molecule drugs targeting these molecules have been developed, and their efficacies are investigated in clinical trials. In addition to aiding the development of novel therapeutics, these clinical studies can inform how critical the targeted molecules are in the regulation of psoriatic inflammation.

This review has summarized the molecular, cellular participants, and signal transduction pathways involved in the immunopathogenic mechanisms of psoriasis that are targeted by the current efforts to develop therapeutics for this disease. Additional mechanisms for modulation of immune responses in psoriasis are continuously to be explored. For example, recent studies have revealed the roles of metabolic pathways, lipid metabolism, and microbiota in the control of skin homeostasis and inflammation [191,192,193]. The exploration of immune modulation mechanisms of psoriasis not only increase our understanding of psoriasis but also accelerate the development of therapeutics for psoriasis.

## Figures and Tables

**Table 1:** Drugs and biologics used for treatment of psoriasis.

Small Molecules	Target/Mechanism	Administration	References
Methotrexate	Increase adenosine production, inhibits lymphocyte function	Oral, S.C.	[124,125,126]
Cyclosporine	Blocking calcineurin-mediated NFTAc dephosphorylation, reducing cytokine production and T cell activation	Oral	[127,128,129]
Acitretin	Oral Retinoid, regulation of keratinocyte proliferation, reducing production of inflammatory cytokines	Oral	[130,131,132]
Fumarate	NF-κB inhibition, anti-oxidation, immunomodulation and anti-inflammation	Oral	[133,134,135]
Apremilast	PDE4 inhibitor, cAMP stabilization, NF-κB suppressing, anti-inflammation	Oral	[136,137,138]
**Biological Agents**	**Target/Mechanism**	**Administration**	**Reference**
Etanercept	Human TNF receptor fusion protein antagonizes TNF-α	S.C.	[139]
Infliximab	Chimeric monoclonal antibody against TNFα	I.V.	[140]
Adalimumab	Fully human monoclonal antibody against TNFα	S.C.	[141]
Certolizumab pegol	monoclonal antibody against TNFα	S.C.	[142,143]
Secukinumab	Human monoclonal antibody against IL-17A	S.C.	[144]
Ixekizumab	Human monoclonal antibody against IL-17A	S.C.	[145]
Brodalumab	Human monoclonal antibody against IL-17 receptor A (IL-17RA)	S.C.	[146,147]
Tildrakizumab	Human monoclonal antibody binds to the p19 subunit of IL-23	S.C.	[148]
Guselkumab	Human monoclonal antibody binds to the p19 subunit of IL-23	S.C.	[149]
Risankizumab	Human monoclonal antibody binds to the p19 subunit of IL-23	S.C.	[150]
Ustekinumab	Human monoclonal antibody binds to the p40 subunit shared by IL-12 and IL-23	S.C.	[151,152]

**Table 2:** Therapeutics currently in the development stages for the treatment of psoriasis.

Biologics	Target	Phase	Administration	References
COVA322	TNF-α/IL-17A antibody fusion protein	I/II (terminated)	I.V.	NCT02243787
BCD-085	Monoclonal antibody binds to IL-17	II (completed)	S.C.	NCT02762994
608	Monoclonal antibody binds to IL-17A	I	S.C.	NCT04367441
MSB0010841	Anti-IL-17A/F nanobody	I (completed)	S.C.	NCT02156466
CJM112	Monoclonal antibody binds to IL-17A/F	I (completed)	S.C.	NCT01828086
M1095	Trivalent monomeric IL-17A/F nanobody	II (completed)	S.C.	NCT03384745
ABT-874	Monoclonal antibody binds to IL-12	II	S.C.	NCT00292396
AK101	IL-12/IL-23 monoclonal antibody	I/II (completed)	S.C.	NCT04172233
Spesolimab	Monoclonal anti-IL-36R antibody	II	I.V.	NCT04399837
Imsidolimab	Monoclonal anti-IL-36R antibody	II (completed)	S.C.	NCT03619902
Anakinra	Recombinant human IL-1R antagonist	II (completed)	S.C.	NCT01794117
Gevokizumab	Monoclonal antibody binds to IL-1β	In clinical development		[157]
Canakinumab	Monoclonal antibody binds to IL-1β	In clinical development		[158]
LY 3471851 (NKTR 358)	PEG conjugate of recombinant human IL-2	I	S.C.	NCT04119557
CC-92252 (DEL 106)	IL-2 mutein Fc fusion protein	I	I.V.	NCT03971825
Abatacept	Fc fusion protein of CTLA-4 ectodomain	II (completed)	I.V.	NCT00287547
LY3462817	Monoclonal antibody to PD-1	I	S.C./I.V.	NCT04152382
CHS-0214	Etanercept biosimilar	III (completed)	S.C.	NCT02134210
MYL-1401A	Adalimumab biosimilar	III (completed)	S.C.	NCT02714322
MSB11022	Adalimumab biosimilar	III (completed)	S.C.	NCT02660580
M923	Adalimumab biosimilar	III (completed)	S.C.	NCT02581345
GP-2017	Adalimumab biosimilar	III (completed)	S.C.	NCT02016105
CHS-1420	Adalimumab biosimilar	III (completed)	S.C.	NCT02489227
MYL-1401A	Adalimumab biosimilar	III (completed)	S.C.	NCT02714322
AVT02	Adalimumab biosimilar	III (completed)	S.C.	NCT03849404
BCD-057	Adalimumab biosimilar	III	S.C.	NCT02762955
Infliximab biosimilar3	Infliximab biosimilar	Recruiting	I.V.	NCT03885089
**Small Molecules**	**Target**	**Phase**	**Administration**	**References**
Dimethyl fumarate	Analogs of Fumaric acid esters	IV	Oral	NCT04263610
ARQ-151	PDE4 inhibitor	II (completed)	Topical	NCT03638258
PF-07038124	PDE4 inhibitor	II	Topical	NCT04664153
Tofacitinib	JAk1/3 inhibitor	II	Oral	NCT04246372
Baricitinib Ruxolitinib	JAk1/2 inhibitor JAk1/2 inhibitor	II II	Oral Topical	NCT01490632 NCT00778700
BMS-986165 Itacitinib (INCB039110) Peficitinib (ASP015 K)	Tyk2 inhibitor JAk1 inhibitor JAk3 inhibitor	III II (completed) II	Oral Oral Oral	NCT04772079 NCT01634087 NCT01096862
Beprocitinib (PF-06700841) PF-06826647	Tyk2/JAK1 inhibitor Tyk2 inhibitor	II (completed) II	Oral and Topical Oral	NCT03895372 NCT03895372
VTP-43742	RoRγT inhibitor	I/II (completed)	Oral	NCT02555709
JET-451	RoRγT inhibitor	II (completed)	Oral	NCT03832738
ABBV-157	RoRγT inhibitor	I (completed)	Oral	NCT03922607
PF-06763809	RoRγT inhibitor	I (completed)	Topical	NCT03469336
CF101	Adenosine A3 receptor agonist	III	Oral	NCT03168256
GSK2982772	Receptor interacting protein kinase 1 (RIPK1) inhibitor	I	Oral	NCT04316585
IMO-8400	TLRs 7, 8, and 9 antagonist	II (completed)	Oral	NCT01899729
CUDC-305	HSP90 Inhibitor	I	Oral	NCT03675542
DMVT-505	Aryl hydrocarbon receptor (AhR) agonist	III	Topical	NCT04053387
Poneslimod	S1P receptor 1 agonist	II (completed)	Oral	NCT00852670
KD025	ROCK2 inhibitor	II (completed)	Oral	NCT02106195

## Data Availability

No new data were created in this review. Data sharing is not applicable to this review article.

## Abbreviations

Tumor necrosis factor alpha (TNF-α); Interleukin (IL); Nuclear factor-kappa-light-chain enhancer of activated B cells (NF-κB); Interferon regulatory factor (IRF); Signal transducer and activator of transcription (STAT); inducible nitric oxide synthase (iNOS); myeloid differentiation factor 88 (MyD88); Janus kinase (JAK); cyclic adenosine monophosphate (cAMP); Psoriasis susceptibility locus (PSORS); Human leukocyte antigen (HLA); A disintegrin-like and metalloprotease domain containing thrombospondin type 1 motif-like 5 (ADAMTSL5); Caspase recruitment domain family member 14 (CARD14); epidermal differentiation cluster (EDC); Angiotensin-converting enzyme (ACE); Dendritic cells (DCs); Langerhans cells (LCs); plasmacytoid DCs (pDCs); myeloid DCs (mDCs); Cluster of differentiation (CD); Blood dendritic cell antigen (BDCA); Toll-like receptor (TLR); Lipopolysaccharide (LPS); Interferon (IFN); macrophage colony-stimulating factor (M-CSF); Colony stimulating factor 1 receptor (CSF-1R); T helper (Th); Regulatory T (Treg); GATA-binding protein 3 (GATA-3); Retineic-acid-receptor-related orphan nuclear receptor gamma T (RORγT); Transforming growth factor beta (TGF-β); forkhead box P3 (FOXP3); Inhibitor of kappaB (IκB); IκB kinase (IKK); cyclooxygenase-2 (COX-2); G protein-coupled receptors (GPCRs); Protein kinase A (PKA); cAMP response element binding protein (CREB); Exchange protein directly activated by cAMP (Epac); Ras-related protein (Rap); Phosphodiesterase 4 (PDE4); Adenylate cyclase (AC); 5′-adenosine monophosphate (5′-AMP); Toll/IL-1 receptor (TIR); IL-1R-associated kinases (IRAKs); TNF receptor-associated factors (TRAFs); transforming growth factor-β-activated kinase 1 (TAK1); TNF receptor (TNFR); Tumor necrosis factor receptor 1-associated death domain (TRADD); Receptor-interacting serine/threonine-protein kinase 1 (RIPK1); IL-17 receptor (IL-17R); RNA-binding proteins (RBPs); Human antigen R (HuR); pre-mRNA-splicing factor SF2 (SF2); Alternative splicing factor 1, AT-Rich interaction Domain 5A (Arid5a); DEAD-Box Helicase 3 X-Linked (DDX3X); C-X-C motif ligand (CXCL); C-C motif ligand (CCL); C-C motif chemokine receptor (CCR); Tyrosine kinase 2 (TYK2); Pathogen-associated molecular patterns (PAMPs); Damage-associated molecular patterns (DAMPs); Antimicrobial peptides (AMPs); ultraviolet (UV); Subcutaneous (S.C.); Intravenous (I.V.); Nuclear factor of activated T cells (NFTAc); Psoriasis Area and Severity Index (PASI); Cytotoxic T-lymphocyte-associated protein 4 (CTLA-4); Programmed cell death protein 1 (PD-1); IL-1R antagonist (IL-1Ra); heat shock protein (HSP); Aryl hydrocarbon receptor (AhR); sphingtosine-1-phosphate receptor 1 (S1PR1); Rho-associated kinase 2 (ROCK2); US Food and Drug Administration (FDA).
